# Supplementary material for: Global Advancement in Pharmacy Services for Mental Health: A Review for Evidence-Based Practices
Source: Healthcare (Basel). 2023 Apr 11;11(8):1082. doi: 10.3390/healthcare11081082 (PMC10137606; doi:10.3390/healthcare11081082)
Supplement: Supplementary file 1 [file healthcare-11-01082-s001.zip › healthcare-2286826-supplementary.doc]

**Table S1: Quality appraisal of the articles included in the review ( R1)**

| **Qualitative studies (n= 5)** | | | | | | |
| --- | --- | --- | --- | --- | --- | --- |
| Study | Criteria | | | | | |
|  | 1.1. Is the qualitative approach appropriate to answer the research question? | 1.2. Are the qualitative data collection methods adequate to address the research question? | 1.3. Are the findings adequately derived from the data? | 1.4. Is the interpretation of results sufficiently substantiated by data? | 1.5. Is there coherence between qualitative data sources, collection, analysis and interpretation? | Sum |
| Hassell, 1997 [18] | Yes | Yes | Yes | Yes | Yes | 5 |
| Tewksbury, 2017 [36] | Yes | Yes | Yes | Yes | Yes | 5 |
| Abrahama, 2017 [53] | Yes | Yes | Yes | Yes | Yes | 5 |
| Mospan, 2020 [67] | Yes | Yes | Yes | Yes | Yes | 5 |
| Wheeler, 2012 [68] | Yes | Yes | Yes | Yes | Yes | 5 |
| **Quantitative randomized control trials (n=14)** | | | | | | |
| Study | Criteria | | | | | |
|  | 3.1. Are the participants representative of the target population? | 3.2. Are measurements appropriate regarding both the outcome and intervention (or exposure)? | 3.3. Are there complete outcome data? | 3.4. Are the confounders accounted for in the design and analysis? | 3.5. During the study period, is the intervention administered (or exposure occurred) as intended? | Sum |
| Losada-Camacho, 2016 [76] | No | Yes | Yes | No | Yes | 3 |
| Mishraa, 2017 [51] | Yes | Yes | Yes | Yes | Yes | 5 |
| Silvia, 2020 [48] | No | Yes | Yes | Yes | Yes | 5 |
| Schmidt, 1998 [46] | Yes | Yes | Yes | Yes | Yes | 5 |
| Liekens, 2014 [42] | Yes | Yes | Yes | Yes | Yes | 5 |
| Valenstein, 2011 [33] | Yes | Yes | Yes | Yes | Yes | 5 |
| Finley, 2003 [32] | Yes | Yes | Yes | Yes | Yes | 5 |
| Al-Saffar, 2008 [30] | Yes | Yes | Yes | Yes | Yes | 5 |
| Aljumah, 2015 [29] | Yes | Yes | Yes | No | Yes | 4 |
| Capoccia, 2004 [26] | Yes | Yes | Yes | Yes | Yes | 5 |
| Crockett, 2006 [27] | Yes | Yes | Yes | Yes | Yes | 5 |
| Brook, 2002 [25] | Yes | Yes | Yes | No | Yes | 4 |
| Adler, 2004 [24] | Yes | Yes | Yes | Yes | Yes | 5 |
| Cochran, 2003 [14] | Yes | Yes | Yes | Yes | Yes | 5 |
| **Quantitative retrospective/ descriptive studies (n=20)** | | | | | | |
| Study | Criteria | | | | | |
|  | 4.1. Is the sampling strategy relevant to address the research question? | 4.2. Is the sample representative of the target population? | 4.3. Are the measurements appropriate? | 4.4. Is the risk of nonresponse bias low? | 4.5. Is the statistical analysis appropriate to answer the research question? | Sum |
| Lauren, 2020 [13] | Yes | No | Yes | No | Yes | 3 |
| Natasa, 2006 [15] | Yes | No | Yes | Yes | Yes | 4 |
| Cochranab, 2017 [16] | Yes | Yes | Yes | Yes | Yes | 5 |
| Bell, 2006 [17] | Yes | Yes | Yes | Yes | Yes | 5 |
| Chong, 2013 [34] | Yes | Yes | Yes | Yes | Yes | 5 |
| Moczygemba, 2011 [35] | Yes | Yes | Yes | Yes | Yes | 5 |
| Bell, 2007 [40] | Yes | Yes | Yes | Yes | Yes | 5 |
| Mathys, 2015 [41] | Yes | Yes | Yes | Yes | Yes | 5 |
| Wang, 2011 [44] | Yes | Yes | Yes | Yes | Yes | 5 |
| Lerat, 2010 [45] | Yes | Yes | Yes | Yes | Yes | 5 |
| Chung, 2011 [49] | Yes | Yes | Yes | Yes | Yes | 5 |
| Moore, 2020 [50] | Yes | Yes | Yes | Yes | Yes | 5 |
| Nishtala, 2009 [54] | Yes | Yes | Yes | Yes | Yes | 5 |
| Castelino , 2010 [55] | Yes | Yes | Yes | Yes | Yes | 5 |
| Gillette, 2020 [66] | Yes | Yes | Yes | Yes | Yes | 5 |
| Rosser, 2013 [64] | Yes | Yes | Yes | Yes | Yes | 5 |
| Gibu, 2017 [73] | Yes | Yes | Yes | Yes | Yes | 5 |
| Leach, 2016 [74] | Yes | Yes | Yes | Yes | Yes | 5 |
| Ilickovic, 2016 [75] | Yes | Yes | Yes | Yes | Yes | 5 |
| Raynsford, 2020 [76] | Yes | Yes | Yes | Yes | Yes | 5 |
| **Mixed-method studies (n=7)** | | | | | | |
| Study | Criteria | | | | | |
|  | 5.1. Is there an adequate rationale for using a mixed-method design to address the research question? | 5.2. Are the different components of the study effectively integrated to answer the research question? | 5.3. Are the outputs of the integration of qualitative and quantitative components adequately interpreted? | 5.4. Are divergences and inconsistencies between quantitative and qualitative results adequately addressed? | 5.5. Do the different components of the study adhere to the quality criteria of each tradition of the methods involved? | Sum |
| O’Reilly, 2014 [65] | Yes | Yes | Yes | Yes | Yes | 5 |
| Knox, 2006 [63] | Yes | Yes | Yes | Yes | Yes | 5 |
| Hare, 2008 [62] | Yes | Yes | Yes | No | Yes | 4 |
| Casey, 2020 [43] | Yes | Yes | Yes | Yes | Yes | 5 |
| Hattingh, 2017 [39] | Yes | Yes | Yes | Yes | Yes | 5 |
| Pauly, 2018 [38] | Yes | Yes | Yes | Yes | Yes | 5 |
| Tallian, 2012 [37] | Yes | Yes | Yes | Yes | Yes | 5 |
| Bell, 2007 [19] | Yes | Yes | No | Yes | Yes | 4 |

**Table S2: Quality appraisal of the articles included in the review (R2)**

| **Qualitative studies (n= 5)** | | | | | | |
| --- | --- | --- | --- | --- | --- | --- |
| Study | Criteria | | | | | |
|  | 1.1. Is the qualitative approach appropriate to answer the research question? | 1.2. Are the qualitative data collection methods adequate to address the research question? | 1.3. Are the findings adequately derived from the data? | 1.4. Is the interpretation of results sufficiently substantiated by data? | 1.5. Is there coherence between qualitative data sources, collection, analysis and interpretation? | Sum |
| Hassell, 1997 [18] | Yes | Yes | Yes | No | Yes | 4 |
| Tewksbury, 2017 [36] | Yes | Yes | Yes | No | Yes | 4 |
| Abrahama, 2017 [53] | Yes | Yes | Yes | Yes | Yes | 5 |
| Mospan, 2020 [67] | Yes | Yes | Yes | No | Yes | 4 |
| Wheeler, 2012 [68] | Yes | Yes | No | Yes | Yes | 4 |
| **Quantitative randomized control trials (n=14)** | | | | | | |
| Study | Criteria | | | | | |
|  | 3.1. Are the participants representative of the target population? | 3.2. Are measurements appropriate regarding both the outcome and intervention (or exposure)? | 3.3. Are there complete outcome data? | 3.4. Are the confounders accounted for in the design and analysis? | 3.5. During the study period, is the intervention administered (or exposure occurred) as intended? | Sum |
| Losada-Camacho, 2016 [76] | No | Yes | Yes | No | Yes | 3 |
| Mishraa, 2017 [51] | Yes | Yes | Yes | Yes | Yes | 5 |
| Silvia, 2020 [48] | Yes | Yes | No | Yes | Yes | 4 |
| Schmidt, 1998 [46] | Yes | Yes | Yes | Yes | Yes | 5 |
| Liekens, 2014 [42] | Yes | Yes | Yes | No | Yes | 4 |
| Valenstein, 2011 [33] | No | Yes | Yes | No | Yes | 3 |
| Finley, 2003 [32] | Yes | Yes | Yes | Yes | Yes | 5 |
| Al-Saffar, 2008 [30] | Yes | Yes | Yes | Yes | Yes | 5 |
| Aljumah, 2015 [29] | Yes | Yes | Yes | No | Yes | 4 |
| Capoccia, 2004 [26] | Yes | Yes | Yes | Yes | Yes | 5 |
| Crockett, 2006 [27] | Yes | Yes | Yes | Yes | Yes | 5 |
| Brook, 2002 [25] | Yes | Yes | Yes | No | Yes | 4 |
| Adler, 2004 [24] | Yes | Yes | Yes | Yes | Yes | 5 |
| Cochran, 2003 [14] | Yes | Yes | Yes | Yes | Yes | 5 |
| **Quantitative retrospective/ descriptive studies (n=20)** | | | | | | |
| Study | Criteria | | | | | |
|  | 4.1. Is the sampling strategy relevant to address the research question? | 4.2. Is the sample representative of the target population? | 4.3. Are the measurements appropriate? | 4.4. Is the risk of nonresponse bias low? | 4.5. Is the statistical analysis appropriate to answer the research question? | Sum |
| Lauren, 2020 [13] | Yes | Yes | Yes | Yes | Yes | 5 |
| Natasa, 2006 [15] | Yes | Yes | Yes | Yes | Yes | 5 |
| Cochranab, 2017 [16] | Yes | Yes | Yes | Yes | Yes | 5 |
| Bell, 2006 [17] | Yes | Yes | Yes | Yes | Yes | 5 |
| Chong, 2013 [34] | Yes | Yes | Yes | Yes | Yes | 5 |
| Moczygemba, 2011 [35] | Yes | Yes | Yes | Yes | Yes | 5 |
| Bell, 2007 [40] | Yes | Yes | Yes | Yes | Yes | 5 |
| Mathys, 2015 [41] | Yes | Yes | Yes | Yes | Yes | 5 |
| Wang, 2011 [44] | Yes | Yes | No | Yes | Yes | 4 |
| Lerat, 2010 [45] | Yes | Yes | Yes | Yes | Yes | 5 |
| Chung, 2011 [49] | Yes | Yes | Yes | Yes | Yes | 5 |
| Moore, 2020 [50] | Yes | Yes | Yes | Yes | Yes | 5 |
| Nishtala, 2009 [54] | Yes | Yes | Yes | Yes | Yes | 5 |
| Castelino , 2010 [55] | Yes | Yes | Yes | Yes | Yes | 5 |
| Gillette, 2020 [66] | Yes | Yes | Yes | No | Yes | 4 |
| Rosser, 2013 [64] | Yes | Yes | Yes | Yes | Yes | 5 |
| Gibu, 2017 [73] | Yes | Yes | Yes | Yes | Yes | 5 |
| Leach, 2016 [74] | Yes | Yes | Yes | Yes | Yes | 5 |
| Ilickovic, 2016 [75] | Yes | Yes | Yes | Yes | Yes | 5 |
| Raynsford, 2020 [76] | Yes | Yes | Yes | Yes | Yes | 5 |
| **Mixed-method studies (n=7)** | | | | | | |
| Study | Criteria | | | | | |
|  | 5.1. Is there an adequate rationale for using a mixed-method design to address the research question? | 5.2. Are the different components of the study effectively integrated to answer the research question? | 5.3. Are the outputs of the integration of qualitative and quantitative components adequately interpreted? | 5.4. Are divergences and inconsistencies between quantitative and qualitative results adequately addressed? | 5.5. Do the different components of the study adhere to the quality criteria of each tradition of the methods involved? | Sum |
| O’Reilly, 2014 [65] | Yes | Yes | Yes | Yes | Yes | 5 |
| Knox, 2006 [63] | Yes | Yes | Yes | Yes | Yes | 5 |
| Hare, 2008 [62] | Yes | Yes | Yes | Yes | No | 4 |
| Casey, 2020 [43] | Yes | Yes | Yes | Yes | Yes | 5 |
| Hattingh, 2017 [39] | Yes | Yes | Yes | Yes | Yes | 5 |
| Pauly, 2018 [38] | Yes | Yes | Yes | Yes | Yes | 5 |
| Tallian, 2012 [37] | Yes | Yes | Yes | Yes | Yes | 5 |
| Bell, 2007 [19] | Yes | Yes | Yes | Yes | Yes | 5 |
